# Supplementary figures and images for: Characterization and comparison of human and mouse milk cells
Source: PLoS One. 2024 Jan 31;19(1):e0297821. doi: 10.1371/journal.pone.0297821 (PMC10830055; doi:10.1371/journal.pone.0297821)

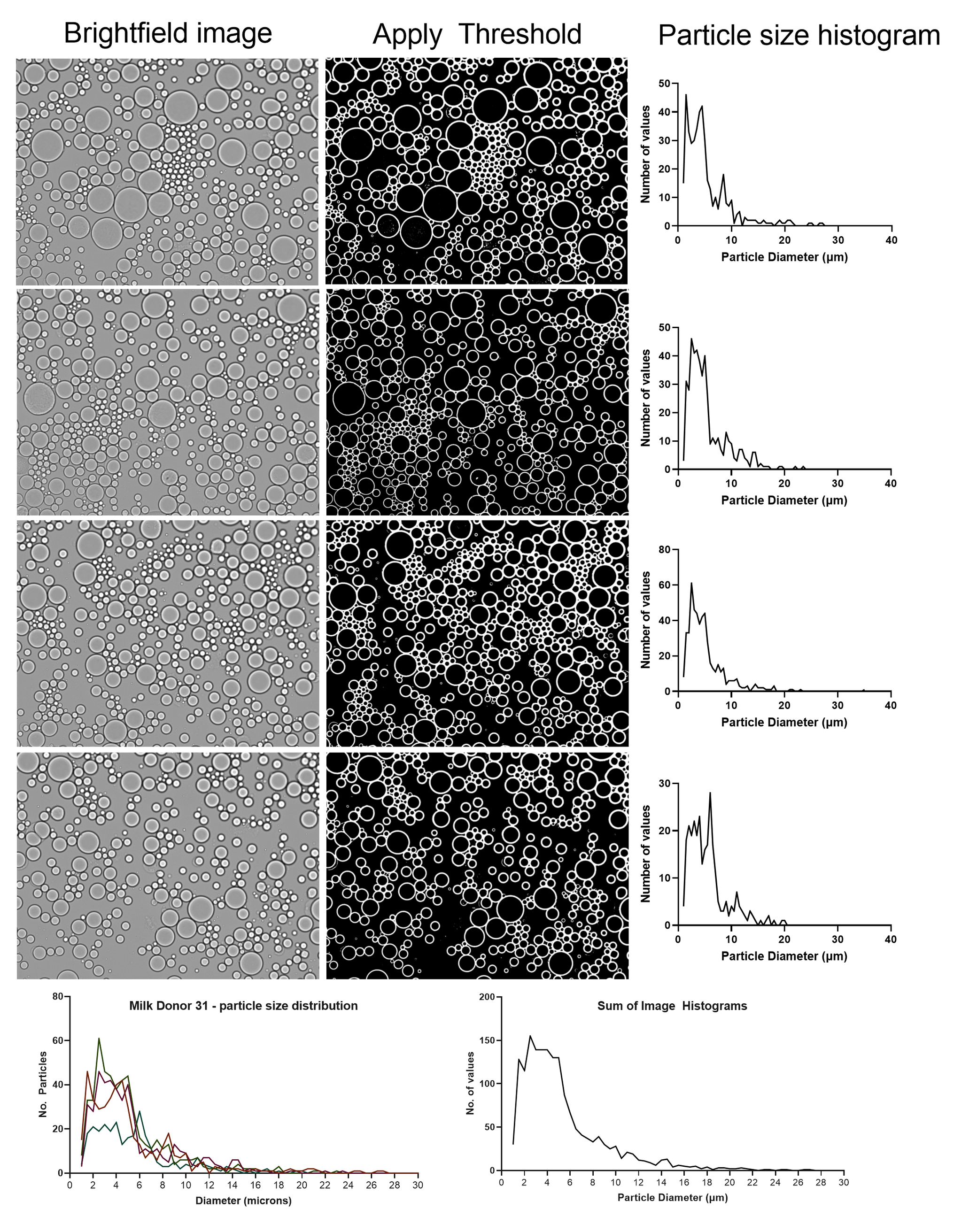

Supplement: S1 Fig — For each brightfield image, a threshold was applied in ImageJ to convert the image into black and white. From these black and white images, particle sizes were measured in microns, and a histogram was generated of the particle sizes. These particle size distributions were added together for 5–6 images per sample of milk. (TIF) [file pone.0297821.s001.tif]
