# Supplementary material for: Characterization and comparison of human and mouse milk cells
Source: PLoS One. 2024 Jan 31;19(1):e0297821. doi: 10.1371/journal.pone.0297821 (PMC10830055; doi:10.1371/journal.pone.0297821)
Supplement: S1 Raw image — (PDF) [file pone.0297821.s002.pdf]

Full blot image annotation for the western blot shown in Fig 6d

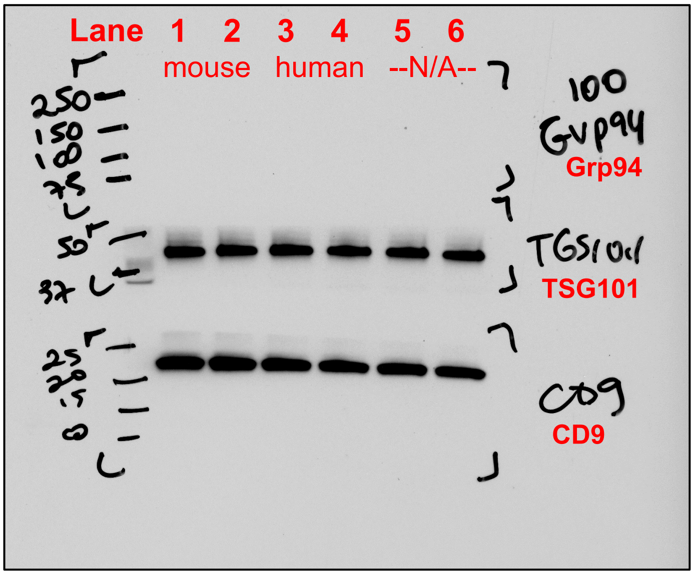

This blot was used to produce Fig 6d. The ladder is shown to the left. Lanes 1-2 and 3-4 correspond to extracellular vesicles isolated from mouse and human milk, respectively. Lanes 5-6 are not applicable to this study and do not appear in the manuscript.

Image was captured with an HP ScanJet Scanner.
