# Supplementary material for: Characterization and comparison of human and mouse milk cells
Source: PLoS One. 2024 Jan 31;19(1):e0297821. doi: 10.1371/journal.pone.0297821 (PMC10830055; doi:10.1371/journal.pone.0297821)
Supplement: Attachment — Submitted filename: plos one report 29 aug 23.docx [file pone.0297821.s003.docx]

The review article entitled “**Characterization and comparison of human and mouse milk cells**” is a nice compilation of the human and mouse milk cells expression at different level like size and age etc.and they secret vesicles. This research study is a summary and emphasis of the human and mouse milk. The experimental designing, writing style and the clarity of the exposition are fine, but the conclusions are not clear. I have some comments for the Author:

**Comment-1** In introduction section author mentioned that they were interested in Extracellular vesicles, what was the reason and how do you decide these are a extracellular vesicles.I could be something else, If they are vesicle what material they are carring inside these vesicles. Have you ever try to find out the material what they are carring like protein /cells/DNA /RNA etc, If these vesicles are same as already establised in the field of different disease then whats a significance of those vesicles.

**Comment-2**

I know milk is a form of fat and cells, Instead of focusing vesicles you should have to focus on immune cells what kind of specialised cells are secreted from both kind of milk. I was curius to know does the only freshsly secreted mouse milk contain maternal cells or they were remain intact in milk in response afer sometime or they dissappear.You also mentioned milk contain membrane enclosed structures in milk, fat globules, and extracellular vesicles, how it could be possible small vesicles containing all together. Is there any scientific way to prove it. I suggest you should have to specify what kind of specialised vesicles are they. I understand author try to diffrentiate b/w milk fat globule in both milk with the help of different stain (fig-3) and you found that mouse milk contained a higher cell density than human milk by three orders of magnitude (Fig 3c) I would like to do suggest some minor experiment to check different Immune cells like T /B, NK, Macrophages cells population in milk cells.

I have some suggestion and queries,

- The abstract section is not well organized and please modify abstract section.
- Author established a procedure to collect milk directly from the mouse mammary gland what kind of procedure you devloped, please mentioned briefly in result section for reader purposes.
- In line no 144 author mentioned that human and mouse milk differ in membrane enclosed structure, did you try to find out what kind of vesicles they were like exosomes apoptosomes what was that ? Please justify.
- In line no 138 Author menbtioned that that milk containg cells are prominent with fat globules that are visually indistinguishable without nuclear staining, did you find out what kind of FAT globules were present or from which cell compartment they arised.
- In Fig -3 FACS figure can you please change the gating starategy as FSC-H/FSC-A And put a rectangle gate on single cell population with the help of polygonal gate. The round gate is not looking good on single cells population for publication quality purposes.
- Fig-4a and in 6a fig resolution is not clear, please change it with good resolution and high brigtness level.
- The authors are advised to change the FACS gating strategy, try to maintain consistency throughout the FACS DATA.
- Conclusion section is poorly written.
- Manuscript can be published but it needs standard review with enriches referencing.
- Authors are strictly advised to first complete the full form then use abbreviations.

This manuscript provides a topic of interest to the researchers in this field and has a potential for possible publication in this journal after some suggested experiments and minor corrections.
